# Evaluation of product of two sigmoidal membership functions (*psigmf*) as an ANFIS membership function for prediction of nanofluid temperature

**DOI:** 10.1038/s41598-020-79293-z

**Published:** 2020-12-18

**Authors:** Meisam Babanezhad, Ali Taghvaie Nakhjiri, Azam Marjani, Mashallah Rezakazemi, Saeed Shirazian

**Affiliations:** 1 Institute of Research and Development, Duy Tan University, Da Nang, 550000 Vietnam; 2 Faculty of Electrical – Electronic Engineering, Duy Tan University, Da Nang, 550000 Vietnam; 3 Department of Petroleum and Chemical Engineering, Science and Research Branch, Islamic Azad University, Tehran, Iran; 4 Department for Management of Science and Technology Development, Ton Duc Thang University, Ho Chi Minh City, Vietnam; 5 Faculty of Applied Sciences, Ton Duc Thang University, Ho Chi Minh City, Vietnam; 6 Faculty of Chemical and Materials Engineering, Shahrood University of Technology, Shahrood, Iran; 7 Laboratory of Computational Modeling of Drugs, South Ural State University, 76 Lenin prospekt, 454080 Chelyabinsk, Russia

**Keywords:** Computational science, Chemical engineering, Mechanical engineering

## Abstract

A nanofluid containing water and nanoparticles made of copper (Cu) inside a cavity with square shape is simulated utilizing the computational fluid dynamics (CFD) approach. The nanoparticles made up 15% of the nanofluid. By performing the simulation, the CFD output is characterized by the coordinates in the x, y, nanofluid temperature, and velocity in the y-direction that these outputs are obtained for different physical time iterations. Moreover, the CFD outputs are examined by one of the artificial techniques, i.e. adaptive network-based fuzzy inference system (ANFIS). For this purpose, the data was clustered via grid partition clustering, and the type of membership functions (MFs) was chosen product of two sigmoidal membership functions (*psigmf*). After reaching 99.9% of intelligence in ANFIS, the nanofluid temperature is predicted for the entire data, which are included in the learning processes. The results showed that the method of ANFIS can predict the thermal properties in different physical times at different computing points without having a training background at those times. Additionally, this study shows that with three membership functions at each input, the model’s accuracy is higher than four functions.

## Introduction

The term nanofluid (NF) refers to a mixture which constitutes solid particle at nano scale dispersed in a liquid. NFs are usually prepared for applications that need fluids with improved transport properties. Over the past years, NF has attracted further attention as a result of its improved features and heat transfer-associated behavior^1,2^, mass transfer^3–6^, moistening and scattering^7^, and antimicrobial activities^8^. Through the nanofluids’ improved thermal behavior, a foundation could be provided for a huge improvement on heat transfer strengthening that is greatly important in some industrial sectors such as transportation, micro-manufacturing, power generation, chemical and metallurgical sectors, oil, and ventilation. Nanofluids are also useful for producing nanostructured materials^9^, complex fluids engineering^10^, and for cleaning oil from surfaces owing to their superb spreading and wetting behavior^11^. In the literature, the nanofluids’ thermal conductivity has been highlighted in the past decade, and within this area it has been focused recently^12–14^.

Numerical evaluation of natural convection in a square shape is a standard investigation as a result of its extensive usage field in engineering, such as cooling the electronic devices. Moreover, in numerous numerical and experimental investigations regarding these geometries, the numerical study is still an attractive research subject to promote the application of nanofluids^15–21^. Nevertheless, further cost and time are crucial for conducting detailed research. To decrease the expense of the investigations for complex systems, soft programming approaches like Artificial Neural Networks (ANNs) or Fuzzy-logic can be recommended to predict heat analysis in the domain^22–24^. However, they are not frequently used to understand the flow and heat problems fully. For instance, the Adaptive Neuro-Fuzzy Inference System (ANFIS)^25^ is also utilized for predicting data in some engineering problems^26^. Nevertheless, it has quite limited usage in energy-related studies. For instance, the ANFIS technique was utilized by Ryoo et al.^27^ for controlling the convergence in fluid simulation. In the study conducted by Lu et al.^28^, ANFIS was utilized for optimizing the in-building heating systems.

Other intelligence-based techniques have been employed for physical systems, such as Fuzzy Inference Systems (FIS) and Fuzzy Logic (FL), which were initially suggested and developed by Zadeh^29^, offering a great tool to make decision in various fields of interest. FL models are represented utilizing IF–THEN rules^30^. Recently machine learning methods stand beside numerical, mathematical, and CFD methods to analyze physical and chemical interactions and thermal distributions in engineering processes. By integrating machine learning methods and numerical algorithms, the optimization of engineering processes is accelerated in terms of computational efforts and expenses. Machine learning methods use numerical results and generate a continuous domain of datasets that accelerate optimization without running expensive numerical methods^26^. They are functioning very fast in terms of learning and prediction processes. Prior works showed that learning time for machine learning methods is very short compared to numerical calculations (e.g. CFD), and the prediction time of machine learning methods can even less than a few seconds which is much shorter than other methods.

Additionally, using machine learning (ML) methods results in avoiding numerical issues and difficulties, such as numerical instability for numerical methods, a convergence of CFD results, the complexity of boundary conditions, meshing geometry, and the creation of high specification mesh in the domain. The ML was used to train local thermal characteristics in the square shape cavity. The results indicated that there was a good agreement between CFD and ML data. In another study, different locations (computing points) of cylindrical bubble column reactors participated in the training method, and flow characteristics, such as velocity components, gas fraction, and turbulence properties were predicted with the ANFIS method. They also used sparger specification as input in the training method and developed a mathematical correlation to predict flow properties as a function of sparger specification^26,31^. In addition to that, several studies concentrated on finding proper model parameters regarding models’ accuracy and prediction capability. They used different membership function specifications (such as number membership functions and type of membership functions) on the model’s accuracy. This type of analysis has been conducted to predict flow properties in the bubble column reactor and thermal properties in the cavity^32^. Several learning algorithms were also used to examine machine learning methods, such as ANNs, ant colony optimization (ACO) algorithms, particle swarm optimization (PSO), and genetic algorithm (GA). Apart from changing learning methods, membership functions, and model parameters, they examined the number of input parameters, number of epoch numbers (numerical iteration), and percentage of training datasets. Their analysis showed that tuning model parameters, and sensitivity study around the number of input, percentage of training datasets, and membership specification should be considered in new datasets and physical processes^26,31^.

In predicting thermal properties in the domain, iterative physical time, computing direction nodes, and velocity distribution in the cavity are considered in the training campaign. This combination of CFD calculated values and CFD input parameters can generate a new way to predict the domain’s temperature. In this regard, the connection and the complexity of input and output parameters are considered in the training framework. This consideration enables researchers to probe into the process and find effective parameters in the process. Apart from different input patterns during training processes, in this research, the product of two sigmoidal membership functions (*psigmf*) function’s impact is considered in translating the training process on the fuzzy interface system for the final decision and prediction framework. Thus, the focus of the current work is to utilize ANFIS for predicting innovative matters based on saving exertion and calculation time in numerical investigations. In the above-mentioned literature, it is obviously shown that few studies exist on analyzing the natural convection through soft computing codes. This study evaluated CFD’s output data using the ANFIS method as one of the artificial intelligence methods. The CFD outputs were simulated by a cavity containing nanofluid with copper nanoparticles. By changing parameters such as the model’s inputs involved in ANFIS method and changing the number of membership functions (MFs), we investigated different conditions for intelligence. With the obtained intelligence, we predicted the nanofluid temperature for different physical time iterations. We predicted that the nanofluid temperature distribution for physical time iteration that was not included in the learning process and was based entirely on the ANFIS prediction capability. The impact of the number of MF and the number of inputs on the model’s accuracy is also considered.

## Mathematical modeling

For the modeling, a square cavity was simulated considering the boundary conditions. Constant-temperature boundary is assumed as the boundary condition for the right and left sides of geometry. Their values without dimensions are equivalent to 1. The right wall consists of less temperature compared to the left side. Adiabatic circumstances are maintained in the top and bottom boundaries for the simulations. NF used here comprises copper (Cu) in H_2_O, and CIP is employed for optimizing the numerical diffusion in this work^23^.

For the modeling, energy and vorticity equations were determined in terms of the dimensionless analysis^33^, where the thermal diffusivity term is expressed as:

1 $$ \alpha_{nf} = \frac{{k_{nf} }}{{(\rho c_{p} )_{nf} }} $$

The detailed description of the model can be found elsewhere^23,33–35^.

## ANFIS model

**Figure 1:**
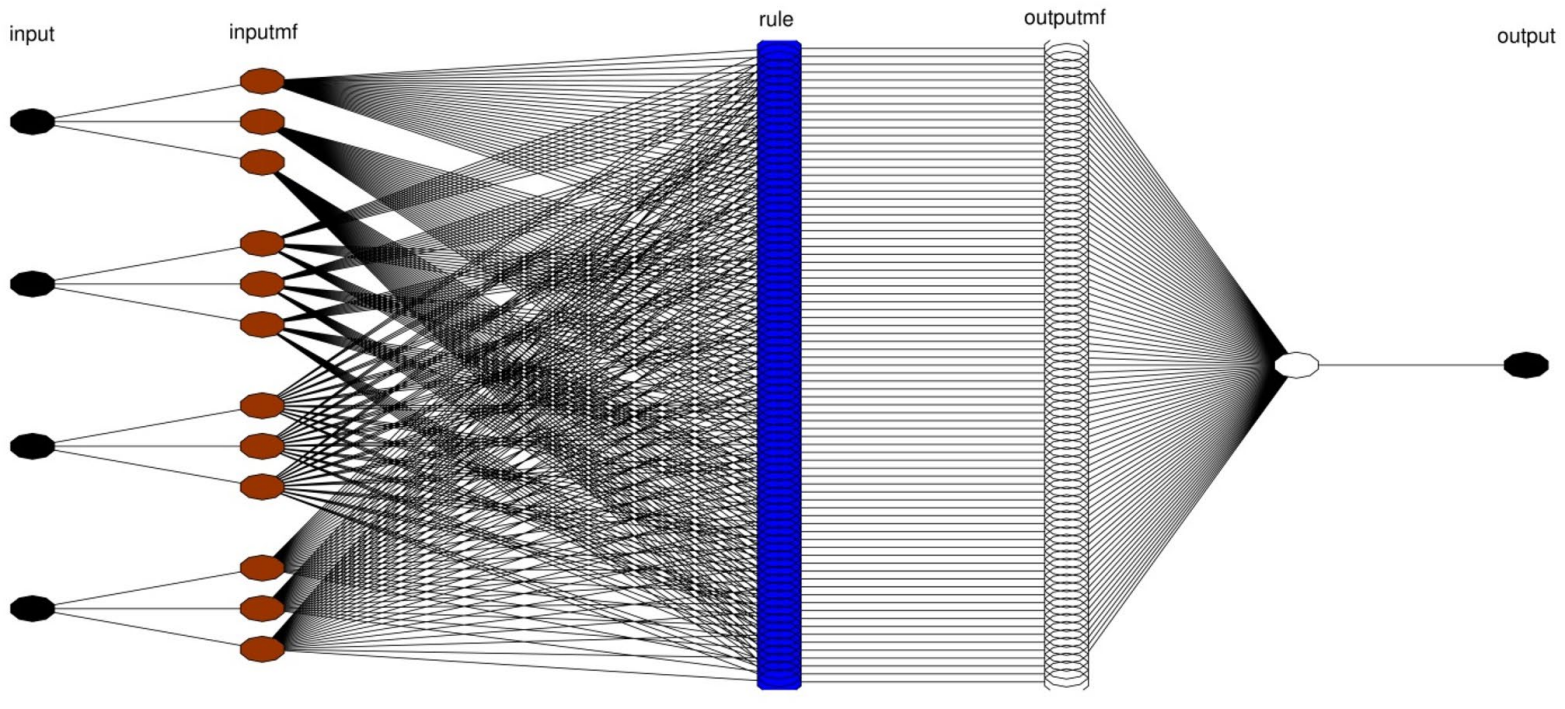
ANFIS structure with four inputs and *psigmf* as MF*.*

ANFIS is classified as a fuzzy inference system that is able to predict the performance of highly complex processes^36^. In ANFIS, Takagi and Sugeno model is mainly employed which is based on if–then rules^23,26,37^. The structure of the utilized ANFIS technique is represented in Fig. 1 to predict the thermal properties in the cavity domain. In this study, (x and y coordinates (x and y computing nodes), nanofluid velocity in the y-direction, and iteration time) are considered to achieve the nanofluid temperature as output. In the first layer of the network’s topology, the inputs split into different numbers of MFs. In this method, ith rule’s function is expressed as^38^:

2 $${s}_{i}=A\left(x\right) B\left(y\right) C\left(v\right) D(time)$$

where *s*_*i*_ represents the outcoming signal of the node of the second layer and *A*, *B*, *C* and *D* stand for the signals incoming from the running MFs on inputs, x-coordinate (*x*), y-coordinate (*y*) and nanofluid velocity in y-coordinate (*v*) and time (time), to the node of the second layer. Detailed explanation of this method has been reported by Takagi and Sugeno^37^.

## Results and discussion

In this study, a square shape was simulated with the CFD method, first. In the domain of cavity, the nanofluid was considered for the acceleration of heat transfer. Temperature distribution in the cavity was based on *x* and *y* computing nodes, velocity distribution (velocity of the fluid in the y-direction), and CFD iteration time was defined as a training dataset for the machine learning method. The whole process of prediction was based on four inputs (velocity distribution, x, y computing nodes, and CFD iteration time) and temperature in the cavity as the output parameter.

To start clustering data, grid partition clustering was used, and membership function (MF) type was also considered as *psigmf* in the model. For another ANFIS setting, the P-value representing % of the training process’s data was considered 70, and the maximum epoch was considered 500.

**Figure 2:**
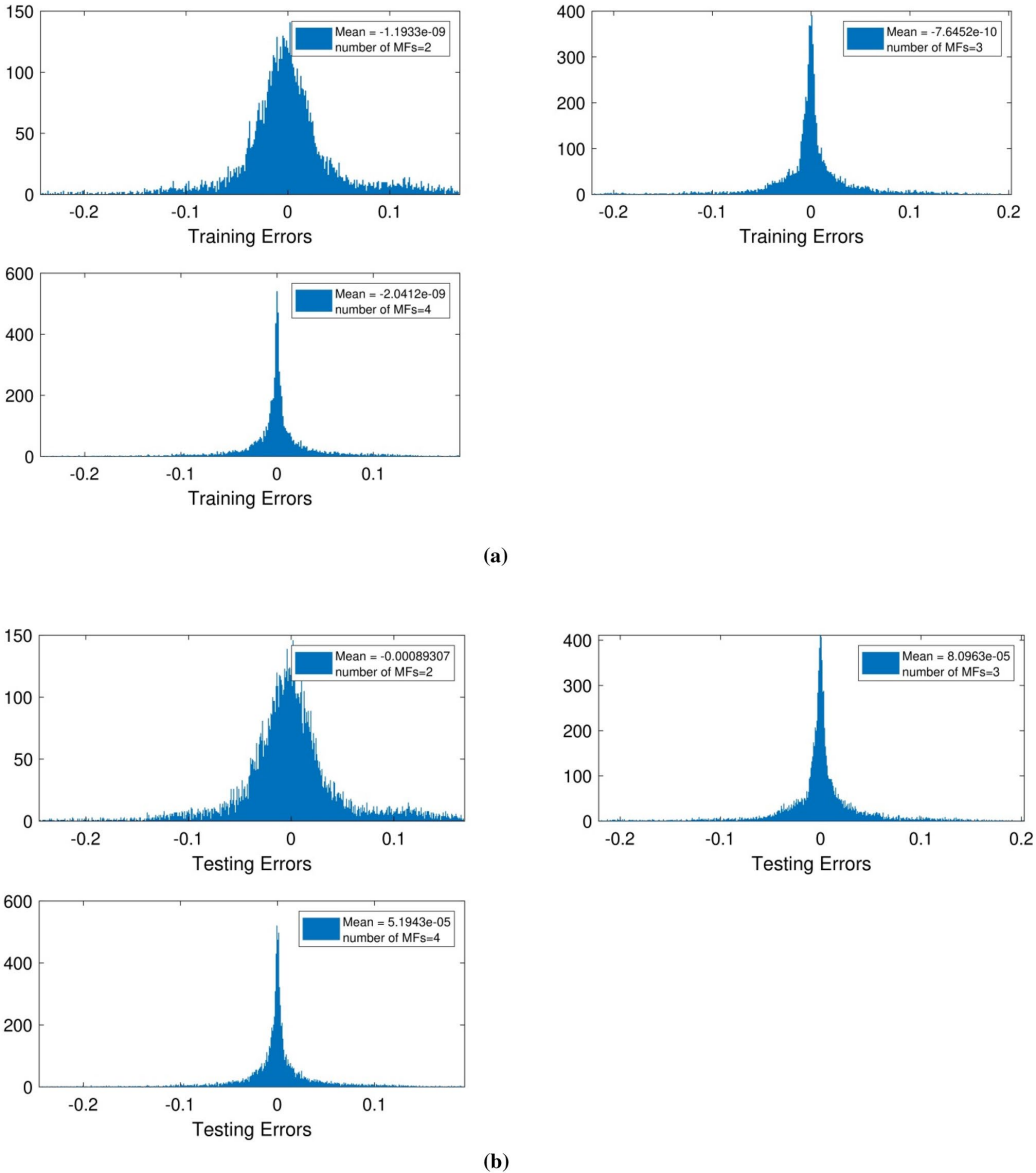
(**a**) Training mean errors with two inputs and *psigmf*. (**b**) Testing mean errors with two inputs and *psigmf*.

Given the assumptions and by incorporating the first and second inputs, which are coordinates in the *x* and *y* directions, the learning process for the number of MFs equal to 2, 3, 4 was performed. Regarding the training process, as can be seen in Fig. 2a with the increasing number of MFs from 2 to 3 and 4, training error inclines more towards zero, and the area under the curve (blue part) decreases. Also, in Fig. 2b which shows the MFs error for the testing process considering different MFs, a decrease in the error value can be seen.

**Figure 3:**
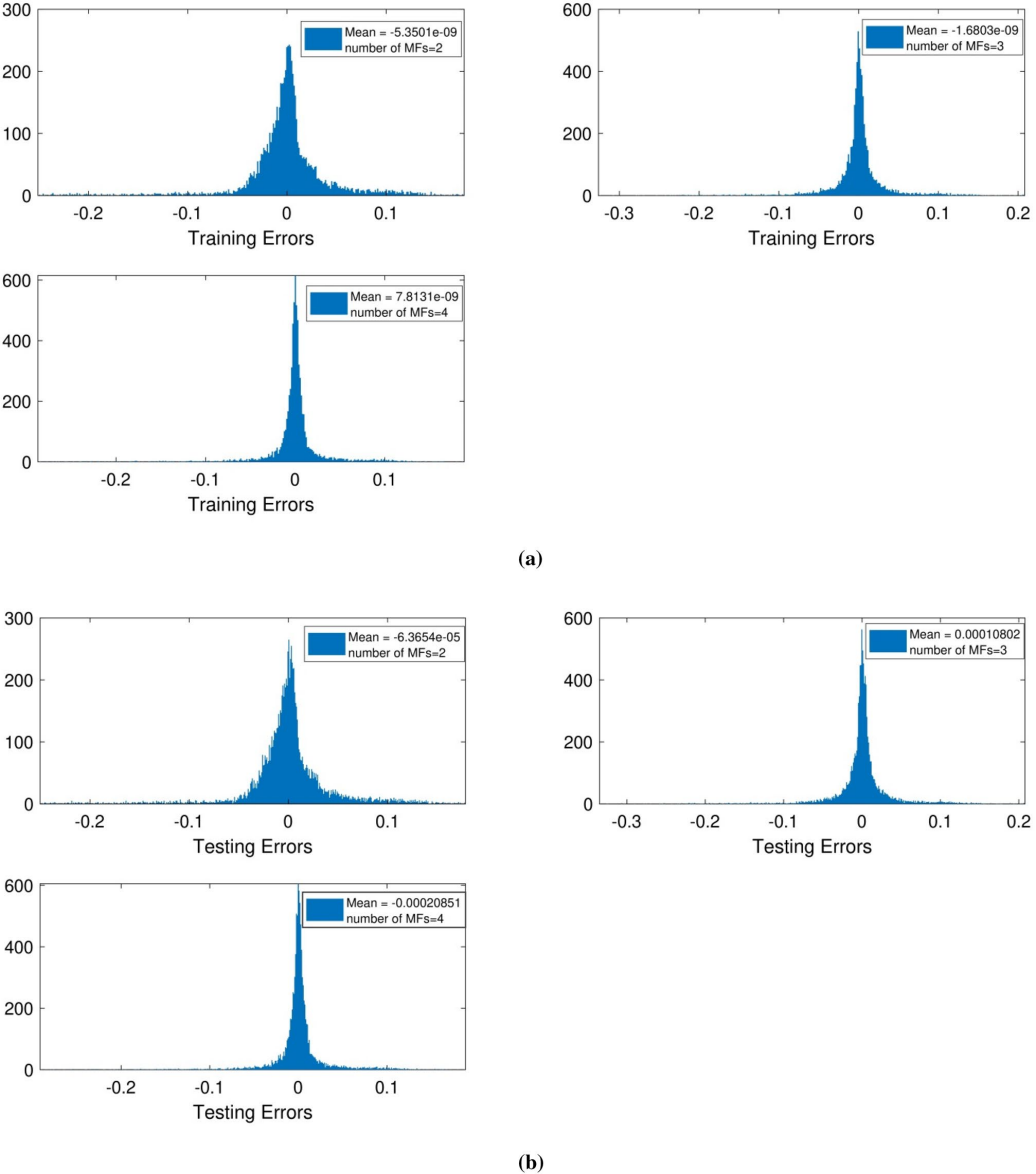
(**a**) Training mean errors with three inputs and *psigmf*. (**b**) Testing mean errors with three inputs and *psigmf*.

To achieve higher prediction capability and model’s accuracy, another ANFIS parameter was changed and fluid speed along y-direction was postulated as third input, and the learning process was repeated again. The results show that number of MFs rose from 2 to 3 with a noticeable change in the system intelligence, but the change in the number of MFs from 3 to 4 in accordance with Fig. 3a,b did not have much effect on ANFIS intelligence. Therefore, iteration was designed in the model as the input 4, and learning process was repeated.

**Figure 4:**
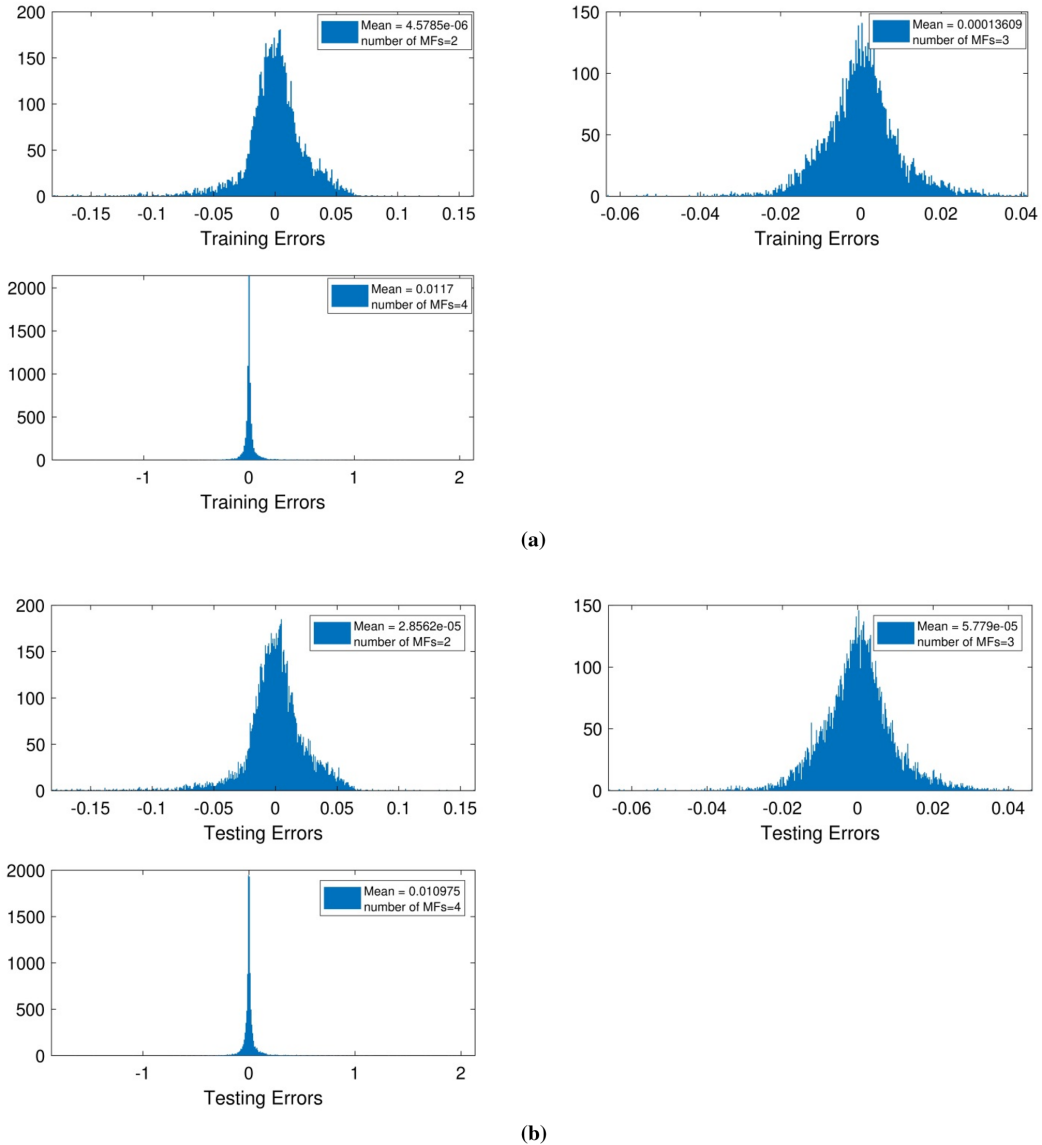
(**a**) Training mean errors with four inputs and *psigmf*. (**b**) Testing mean errors with four inputs and *psigmf*.

Figure 4a,b show that the error range compared with a similar situation in terms of using three inputs had a decent decline and increased the number of MFs from 2 to 3.

**Figure 5:**
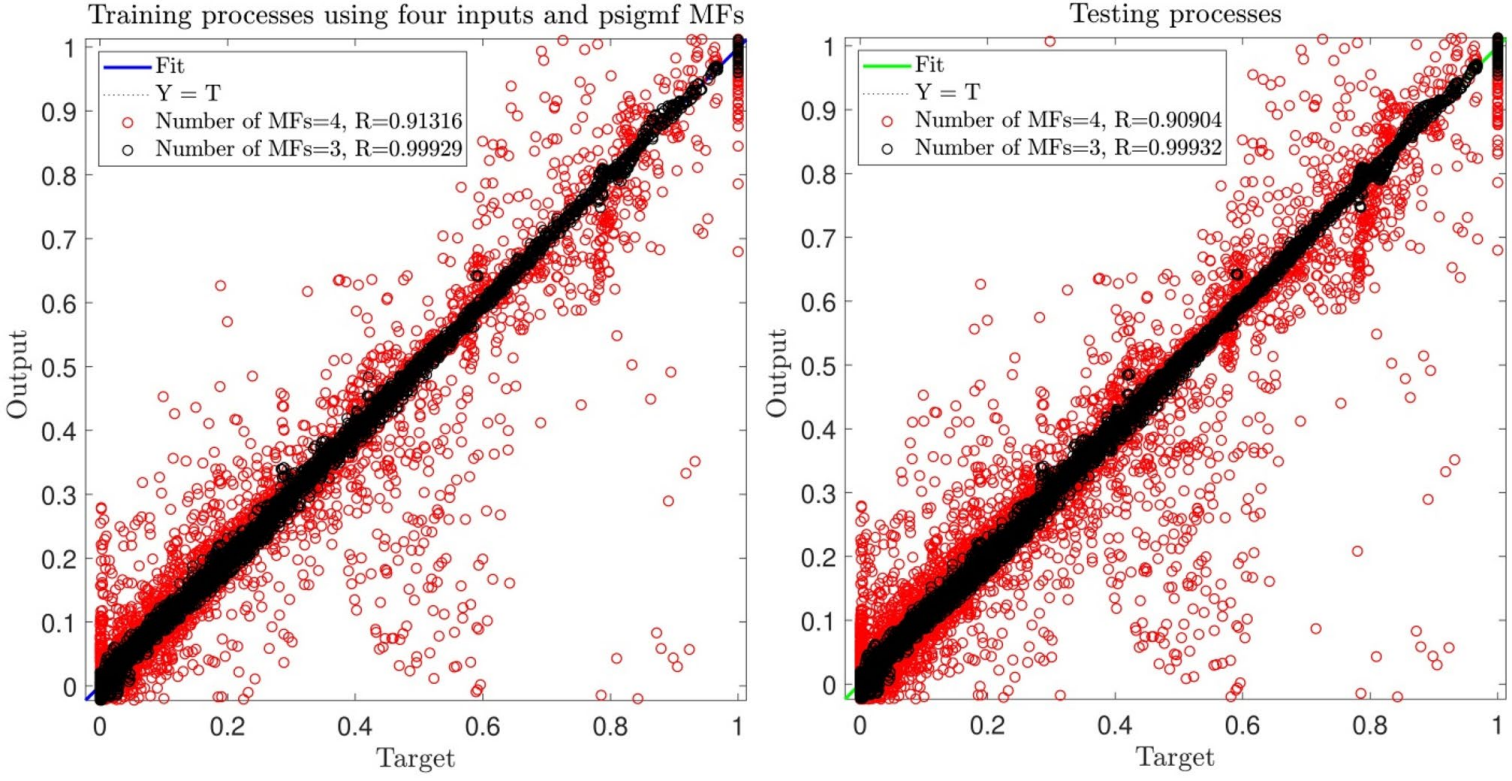
Training and testing with four inputs and *psigmf*.

By comparing ANFIS output, which is the CFD output of nanofluid temperature, the ANFIS target (nanofluid temperature) is observed that is predicted by ANFIS. It is observed that we have reached to 99.9% in testing processes with three membership functions. In this analysis, the impact of two different numbers of MFs in each input was examined on the model’s ability. The outcomes showed that three membership functions enhance the accuracy of the model, while by the increasing number of functions to four, there is a divergence in model behavior. Therefore, these results showed that the increment of the functions could not guarantee a high level of accuracy or prediction capability (Fig. 5).

**Figure 6:**
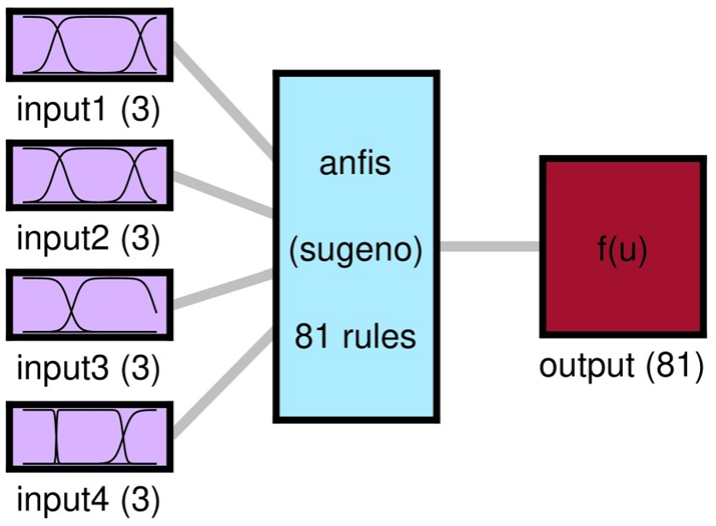
FIS structure with four inputs and *psigmf*.

**Figure 7:**
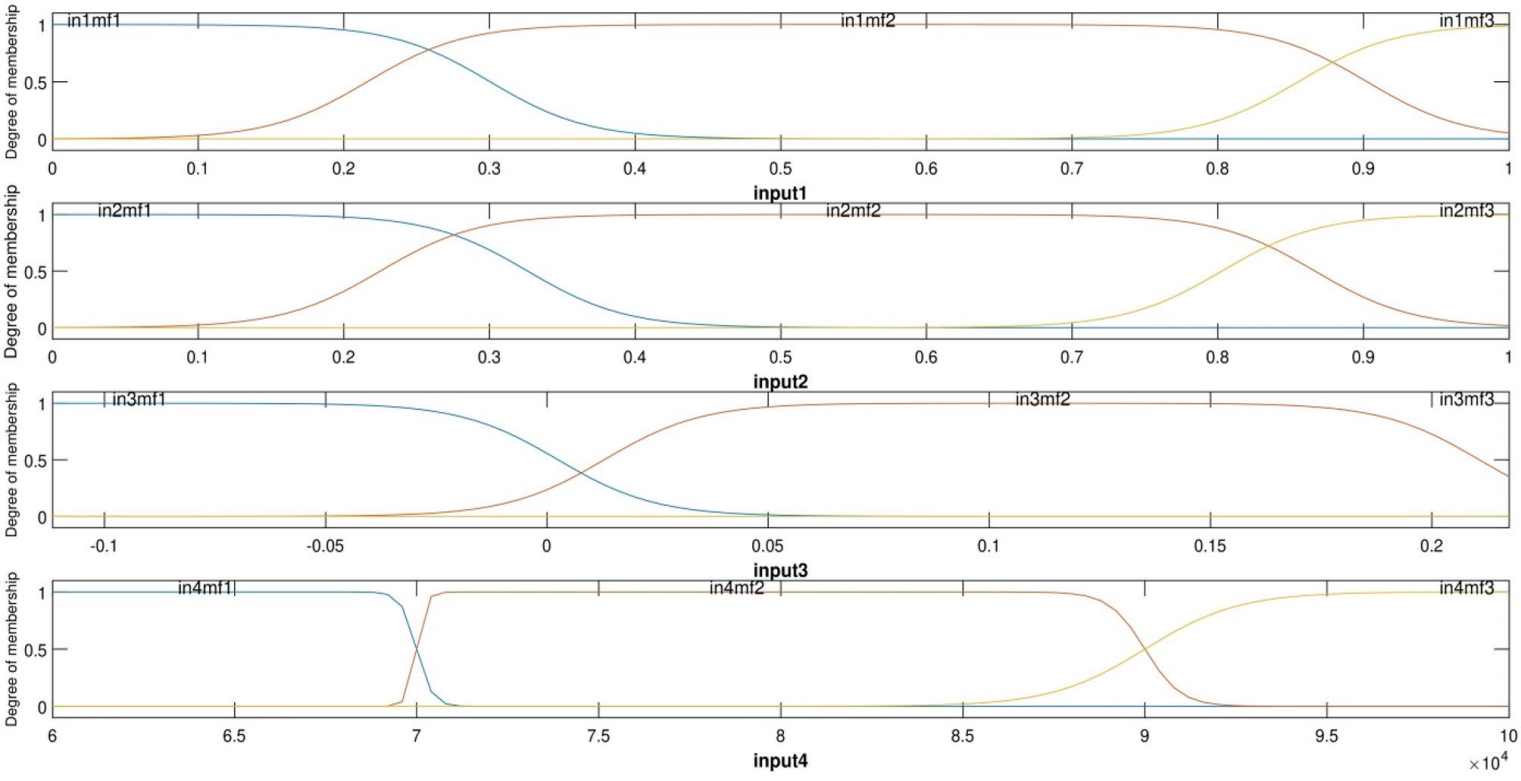
Degree of membership with four inputs and *psigmf*.

**Figure 8:**
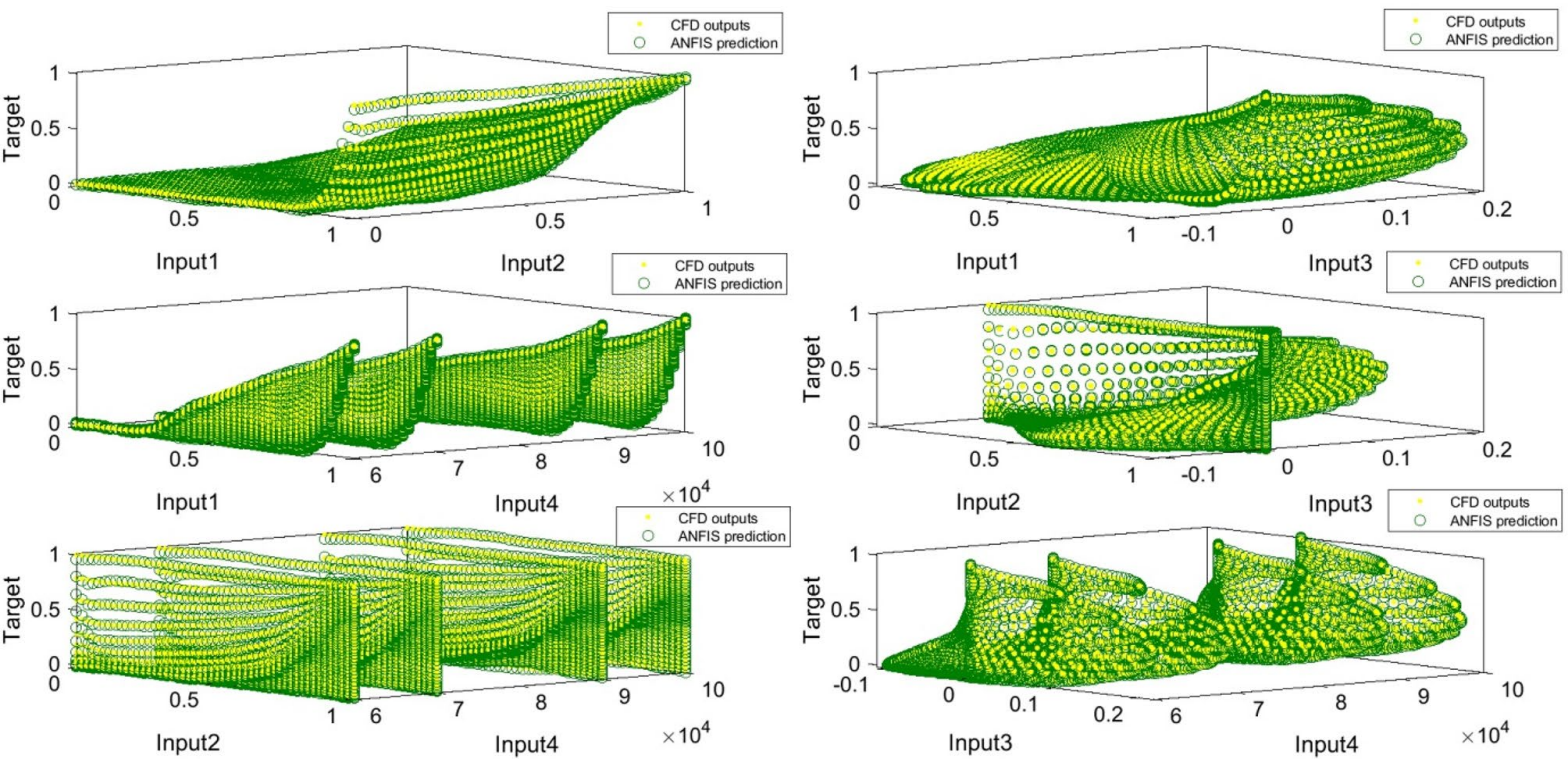
ANFIS validation with various inputs and *psigmf*.

To achieve a high amount of intelligence according to Fig. 6, four inputs with the number of MFs equal to 3, the type of MFs was *psigmf* and the number of rules created by ANFIS was 81 rules. In Fig. 7, four inputs and 3 MFs are *psigmfs*. The ANFIS intelligence can predict different points of the cavity. As shown in Fig. 8, the matching between CFD output points and ANFIS predictions is quite evident, confirming the validity of the designed ANFIS structure. As shown in Fig. 8, there was an excellent agreement between CFD and ANFIS results for different input parameters.

**Figure 9:**
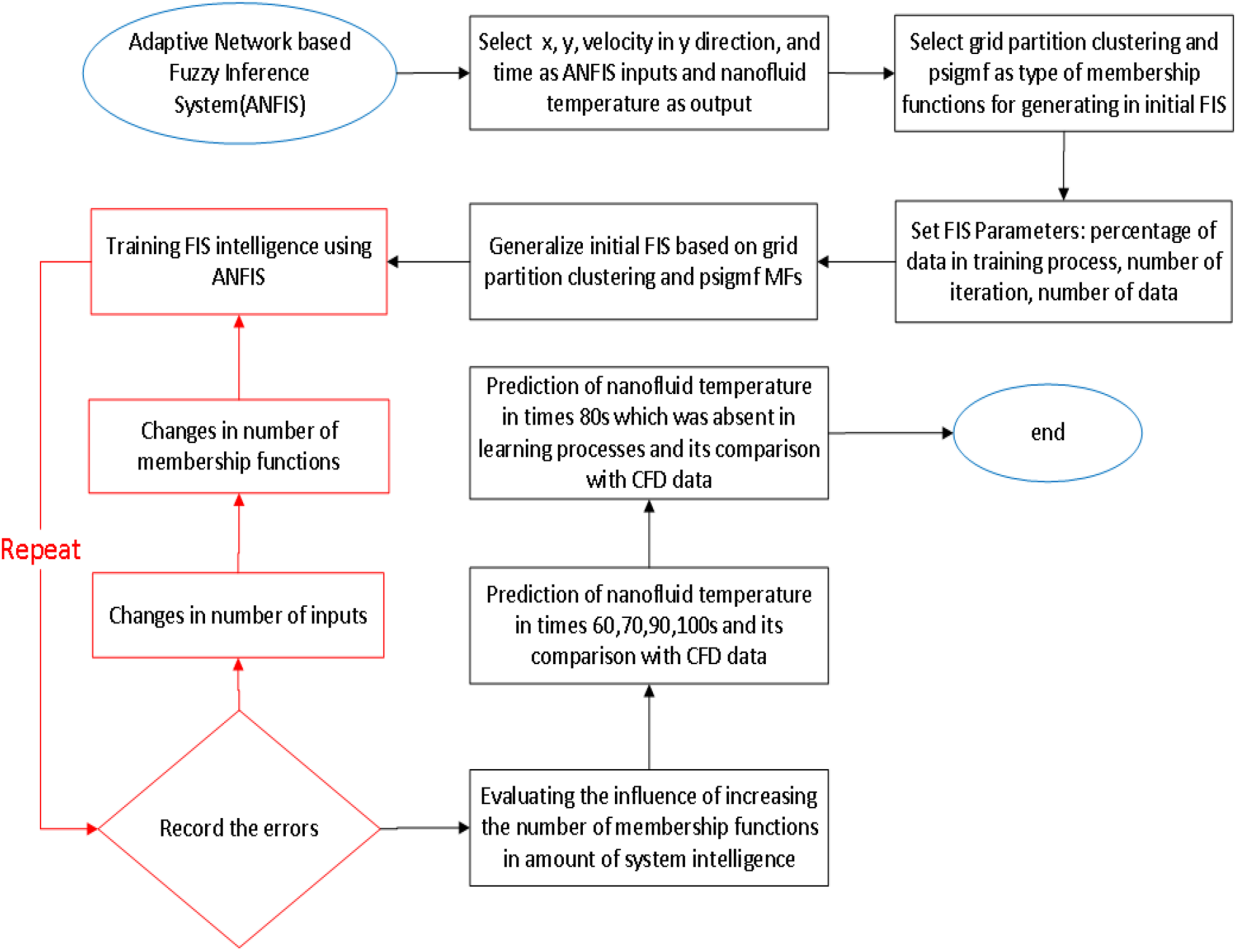
Flowchart for the selection of model parameters and prediction steps.

**Figure 10:**
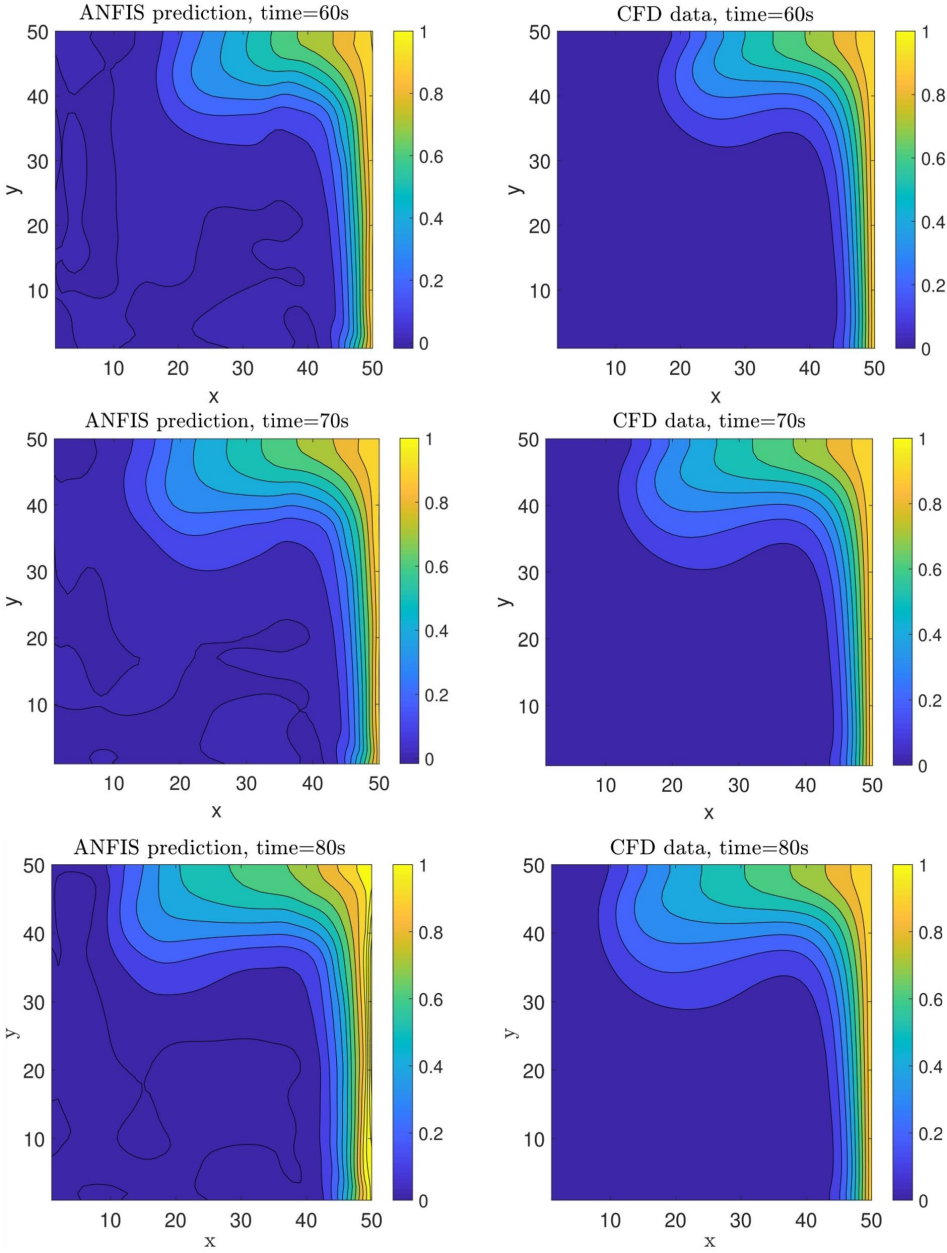

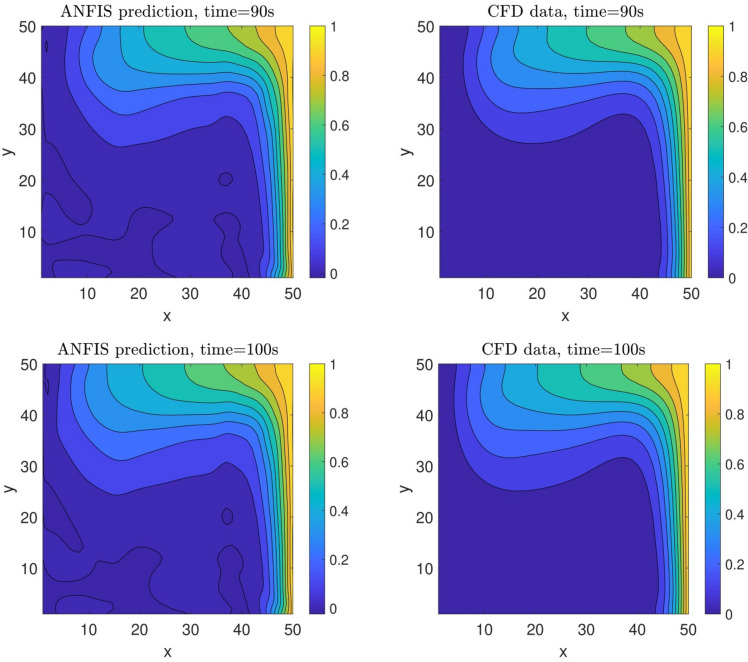
Prediction of nanofluid temperature at different times.

To better understand the model implementation (selection of model parameters and inputs) and the ability of model prediction in terms of evaluation algorithm, the flow chart of model implementation is fully illustrated in Fig. 9. In selecting input parameters, *x*, *y* computing points (nodes), velocity distribution, and CFD iteration time are considered as input parameters, while the output of the model is thermal distribution. In the next step, the grid partition clustering along with *psigmfs* is defined in the model. Additionally, the number of epoch or numerical iteration, percentage of training data, and the numbers of data are considered in the third level of defining the model parameters. Then, the initial FIS structure is generated based on the definition of grid partition clustering and *psigmf*. In the next step, the training of the FIS structure with the ANFIS method is started. However, to assess the model’s accuracy, the model’s error is considered in the algorithm. If the high value of error is recorded in the algorithm, the number of inputs and membership functions are changed. After passing error assessment and finding the final and proper membership function to predict the temperature in the cavity, the ANFIS method predicts thermal distribution for different time (60, 70, 90, and 100 s). These results are also compared with CFD results. However, they participated in the training processes at the beginning. To fully evaluate the model, the ANFIS method is called a model for 80 s iteration time. In this regard, the machine learning algorithm can predict the results that the ANFIS method did not train in the previous steps. Figure 10 shows the distribution of temperature as a function of *x* and *y* computing nodes. The results show that the ANFIS method could correctly track the domain’s temperature, and the results are in good agreement with CFD results. In the beginning, this examination is for training time, and all datasets participated in the training method. However, to test the prediction capability, the ANFIS method also predicts the non-train time, a well. The results showed that there is also a great agreement between CFD and the ANFIS method in terms of thermal distribution in the domain, particularly at the right-hand side of the cavity domain. For a better prediction of the thermal and flow characteristics in the cavity domain at the left-hand side, more datasets are required at the boundary conditions.

**Figure 11:**
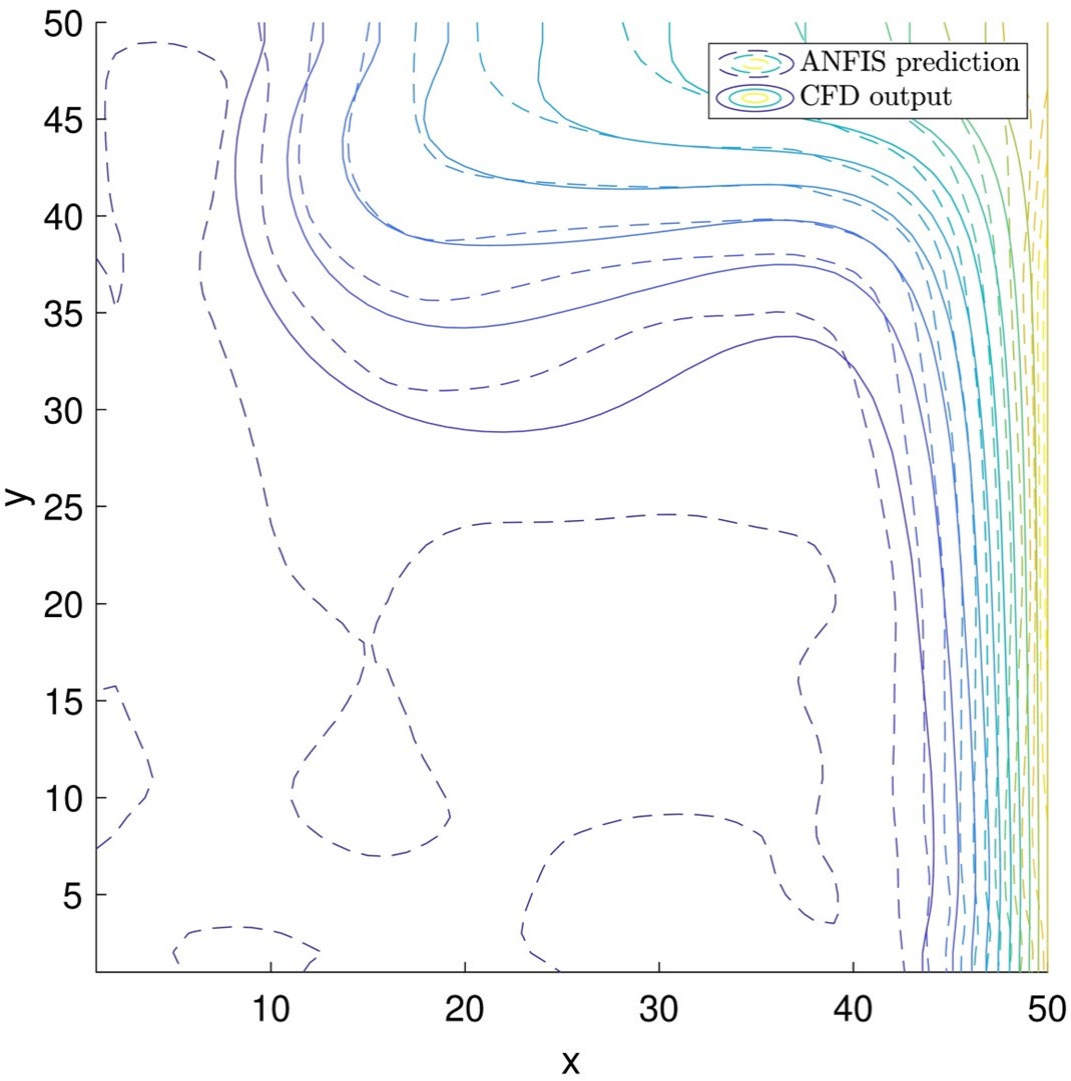
Comparison of nanofluid temperature contour between ANFIS prediction and CFD output.

For a better comparison between the CFD and the ANFIS method results, the results of two different methods are also plotted against each other (Fig. 11). The figure shows that the method of ANFIS can show the thermal distribution from the right-hand to the left-hand side. These results are in good agreement with CFD results. However, very close to the left solid walls, there is a marginal difference between the two different methods. In this regard, the ANFIS method at the left solid walls shows a lower temperature than the CFD method.

## Conclusion

In this study, the two-dimensional cavity containing nanofluid was simulated with the CFD. Then, fluid characteristics such as temperature and velocity distributions as a function of *x* and *y* computing nodes and CFD iterations, were considered as a training dataset in the machine learning framework, ANFIS method. However, the temperature distribution in the domain is considered as the output of the model. Two different processes were considered for the assessment of the machine learning method, such as training and testing framework. This method contained a high level of accuracy and prediction capability in the training and testing with R > 0.9. The results showed that the machine learning approach could accurately predict the process with high prediction ability. In this regard, the machine learning method can accurately track the temperature distribution in the cavity domain with similar behavior as the CFD calculation. However, there were some differences near CFD boundary conditions. To improve this minor numerical discrepancy, more CFD data set is required for the training process of the machine learning method, or dataset filtration is necessary near the boundary conditions. The ANFIS results also show that increment of the number of membership functions cannot guarantee the improvement of the model in terms of model’s accuracy and prediction capability. This method is also a capable tool to track temperature distribution for different physical times in the cavity domain without any information or training background.

For further studies, machine learning methods can be a great option to train the flow characteristics in the cavity as a function of physical time with different learning methods (GA, ACO, and PSO) to understand better the process and development of more reliable prediction tools in engineering processes. Prediction of the different regimes of heat and mass transfer in the cavity can be defined as the main limitation of the current study and a combination of numerical and machine learning methods. The machine learning method cannot estimate the change of flow property due to geometry differences or different operating conditions that explain different physics (not in the training process). Additionally, learning big data requires parallel computing and high specification cloud computing. To improve the model for faster learning and prediction, dimension of datasets should be normalized based on significant process parameters. This combination can be used for an unpredicted environment in the engineering process, and it can be defined as a game-changer in the modeling area.
